# A single‐centre, real‐world study of BTK inhibitors for the initial treatment of MYD88^mut^ /CD79B^mut^ diffuse large B‐cell lymphoma

**DOI:** 10.1002/cam4.7005

**Published:** 2024-03-08

**Authors:** Ting Deng, Shiyuan Zhang, Min Xiao, Jia Gu, Liang Huang, Xiaoxi Zhou

**Affiliations:** ^1^ Department of Hematology Chongqing Fifth People's Hospital Chongqing PR China; ^2^ Department of Hematology Tongji Hospital, Tongji Medical College, Huazhong University of Science and Technology Wuhan Hubei PR China

**Keywords:** Bruton's tyrosine kinase inhibitor, CD79B^mut^, diffuse large B‐cell lymphoma, MCD subtype, MYD88^mut^

## Abstract

**Background:**

MCD (MYD88^L265P^/CD79B^mut^) diffuse large B‐cell lymphoma has a poor prognosis. There is no published clinical research conclusion regarding zanubrutinib or orelabrutinib for the initial treatment of MCD DLBCL.

**Aims:**

This study aimed to analyse the efficacy and safety of Bruton's tyrosine kinase inhibitor (BTKi) (zanubrutinib or orelabrutinib) therapy for newly diagnosed DLBCL patients with MYD88^mut^ and/or CD79B^mut^.

**Materials and Methods:**

Twenty‐three newly diagnosed DLBCL patients with MYD88^mut^ and/or CD79B^mut^ from June 2020 to June 2022 received BTKi combined with rituximab plus cyclophosphamide, doxorubicin, vincristine and prednisone (R‐CHOP) or rituximab + lenalidomide (R^2^). A control group of 17 patients with MYD88^mut^ and/or CD79B^mut^ DLBCL who received the standard R‐CHOP therapy was also assessed. We retrospectively analysed clinical characteristics, safety, overall response rate (ORR), complete response (CR) rate and progression‐free survival (PFS) of the two groups.

**Results:**

The main clinical features were a high International Prognostic Index (IPI) score (≥3, 22/40, 55%) and a high rate of extranodal involvement (27/40,67.5%). Among the 23 DLBCL patients, 18 received BTKi + R‐CHOP, and five elderly DLBCL patients were treated with BTKi + R^2^. Compared with those in the control group (ORR 70.6%, CRR 52.9%, 1‐year PFS rate 41.2%), improved ORR, CRR and PFS results were observed in the BTKi + R‐CHOP group (100%, 94.4% and 88.9%, *p* = 0.019, 0.007, and 0.0001). In subgroup analyses based on genetic subtypes, cell origin, dual expression or IPI score, patients in the BTKi + R‐CHOP group had better PFS than patients in the control group. In the BTKi + R‐CHOP group, no significant difference was found in ORR, CRR and PFS based on subtype analysis, while BTKi‐type subgroups exhibited statistically significant differences in 1‐year PFS (*p* = 0.028). There were no significant differences in grade 3–4 haematological toxicity (*p* = 1) and grade 3–4 non‐haematological toxicity (*p* = 0.49) between the BTKi + R‐CHOP and R‐CHOP treatment groups. In the BTKi + R^2^ group, the ORR was 100%, the CRR was 80%, and the 1‐year PFS rate was 80%. The incidences of grade 3–4 haematologic toxicity and non‐haematological toxicity were both 40%. No bleeding or cardiovascular events of grade 3 or higher occurred in any patients.

**Discussion:**

The efficacy of BTKi combined with R‐CHOP was similar to previous reports, which was significantly better than R‐CHOP alone. It is necessary to fully consider that 14 patients in the BTKi + R‐CHOP group received a BTKi as maintenance therapy when evaluating efficacy. Meanwhile, the addition of a BTKi may improve the prognosis of non‐GCB, DEL or high‐IPI‐score DLBCL patients with MYD88^mut^ and/or CD79B^mut^. In our study, five elderly DLBCL patients with MYD88^mut^ and/or CD79B^mut^ were achieved better ORR, CRR, PFS than the historical data of R‐miniCHOP treatment and Ibrutinib + R^2^ treatment. However, the efficacy and benefit of BTKis for this type of DLBCL need to be further analysed using a larger sample size.

**Conclusion:**

This study suggests that newly diagnosed DLBCL patients with MYD88^mut^ and/or CD79B^mut^ may benefit from BTKis according to real‐world clinical data.

## 1 INTRODUCTION

Diffuse large B‐cell lymphoma (DLBCL) is the most common histological subtype of aggressive lymphoma. The molecular typing of DLBCL has been conducted in multiple studies, and the MYD88^L265P^ and CD79B^mut^ subtypes (MCD subtypes) have a poor prognosis. MCD subtypes tend to involve extranodal organs, mainly ABC‐type DLBCL, and the 3‐year event‐free survival (EFS) and 3‐year overall survival (OS) after rituximab plus cyclophosphamide, doxorubicin, vincristine, and prednisone (R‐CHOP) treatment are 48% (*p* = 0.01) and 69.6% (*p* = 0.032),^1^, ^2^ respectively. In addition to MCD subtypes, increasingly more studies indicate that MYD88^L265P^CD79B^wt^ and MYD88^wt^CD79B^mut^ are also associated with poorer prognosis.^3^, ^4^, ^5^, ^6^ It has been reported that the 5‐year OS of patients with MYD88^L265P^ is 52%.^7^ Although there is no significant difference in OS between CD79B^mut^ DLBCL patients and CD79B^wt^ patients, the cumulative incidence of recurrence/progression is significantly increased (56.3% vs. 35.1%, *p* = 0.019).^6^

A large number of studies have shown that MCD DLBCL is extremely dependent on the B‐cell receptor signalling pathway and that blocking Bruton's tyrosine kinase (BTK) can effectively interfere with this pathway, thereby achieving an anti‐lymphoma effect.^8^, ^9^, ^10^ Clinical studies have confirmed that both ibrutinib and zanubrutinib can achieve good overall response rates (ORRs) in patients with relapsed/refractory (R/R) MCD DLBCL (zanubrutinib 50%, 3/6^11^; ibrutinib 80%, 4/5^12^). A subgroup analysis in the PHOENIX study^13^ showed that the 3‐year EFS and OS of young patients (age <60 years, *n* = 31) with MCD DLBCL treated with ibrutinib and R‐CHOP were both 100%, significantly better than those for controls in previous studies.^14^ At present, there is no published clinical research conclusion regarding zanubrutinib or orelabrutinib for the initial treatment of MCD DLBCL, and there is also a lack of real‐world reports.

The overall prognosis is worse for elderly patients with lymphomas than for younger patients with lymphomas, owing to poor tolerance to treatment,^13^, ^15^ especially for those with high‐risk molecular prognostic factors, such as MCD subtypes. Such patients are more in need of low‐toxicity and high‐efficiency targeting strategies to improve prognosis. There have been clinical studies using the IR^2^ (ibrutinib, rituximab and lenalidomide) regimen, and good curative effects were obtained, of which 1 CD79B^mut^ patient was in continuous remission for more than 3.9 years.^16^ However, there are no reports on the use of this regimen in elderly patients with MCD DLBCL. Preclinical studies have shown a synergistic effect between lenalidomide and BTK inhibitors (BTKis).^9^ Therefore, the chemo‐free regimen of a BTKi combined with *R* ^2^ may represent a safer and more effective treatment option for elderly patients with MCD DLBCL. In view of these findings, the Chinese Society of Clinical Oncology (CSCO) guidelines for the diagnosis and treatment of malignant lymphoma (2023) indicate that BTKi + R‐CHOP may improve the survival of treatment‐naive patients with the aforementioned subtypes of DLBCL (e.g. MCD, N1, non‐GCB and double expresser).^17^

This is a report about a real‐world study of the safety and efficacy of combined BTKi therapy as an initial treatment for 23 patients with MYD88^mut^ and/or CD79B^mut^ DLBCL.

## 2 MATERIALS AND METHODS

### 2.1 Patient characteristics

The curative effect and efficacy of BTKi (orelabrutinib or zanubrutinib) therapy were retrospectively assessed in 23 patients with newly diagnosed DLBCL treated at the Department of Haematology, Tongji Hospital, Tongji Medical College of Huazhong University of Science and Technology, from June 2020 to June 2022. A control group of 17 patients with MYD88^mut^ and/or CD79B^mut^ treated with the standard R‐CHOP therapy was also included. This study was approved by the Medical Ethics Committee of Tongji Hospital, Tongji Medical College of Huazhong University of Science and Technology, in accordance with the Declaration of Helsinki. The patients in this study gave their written informed consent in accordance with the Declaration of Helsinki. All patients were pathologically diagnosed by lymph node biopsy,^18^ and cell of origin was identified using Han's criteria.^19^ All patients underwent FDG PET/CT and bone marrow examination to evaluate the tumour stages and treatment efficacy. Patients with risk factors for central nervous system involvement also received cerebrospinal fluid examination. These patients had MYC ≥40% and BCL2 ≥50%, defined as double‐expressing lymphoma (DEL).^20^ The clinical characteristics and laboratory data of patients, including age, sex, stage (Lugano 2014 classification), International Prognostic Index (IPI) score,^21^ Eastern Cooperative Oncology Group (ECOG) grade,^22^ lactate dehydrogenase, etc. were collected from the electronic medical record system. Genomic DNA was extracted from tumour tissues. The hotspot mutations in DLBCL (including TP53, ARID1A, B2M, BTG1, BTG2, CARD11, CCND3, CD70, CD79B, CIITA, CREBBP, DDX3X, DTX1, DUSP2, EP300, EZH2, FAS, GNA13, IRF4, IRF8, KMT2D, MPEG1, MYD88, NOTCH1, NOTCH2, PIM1, PRDM1, SGK1, SOCS1, STAT3, STAT6, TBL1XR1, TET2, TNFAIP3, TNFRSF14, ZFP36L1, etc.) were assessed using next‐generation sequencing (NGS) with NEXTSEQ550 (Illumina, SanDiego, CA, USA). Fluorescence in situ hybridization (FISH) was used to detect BCL2, BCL6 and MYC rearrangement. Based on NGS and FISH results, the MCD‐like, ST2‐like, BN2‐like, TP53mut subtypes were defined using the LymphPlex classification tool.^23^ The last follow‐up was on June 30, 2023.

### 2.2 Treatment options and evaluation of efficacy

Eighteen patients received covalent BTKis (eight received zanubrutinib and ten received orelabrutinib) combined with the R‐CHOP regimen; five elderly patients received covalent BTKis (three received zanubrutinib and two received orelabrutinib) combined with the *R* ^2^ regimen; and 17 patients received the R‐CHOP regimen. The specific regimens are detailed in Table S1. Thirty‐eight patients received six sessions of treatment. However, two elderly patients in the BTKi + *R* ^2^ group discontinued the induction therapy after four sessions of treatment due to COVID‐19 infection. All 23 patients started to receive BTKi after the molecular subtype was determined based on the NGS results in the second session of treatment. Fifteen patients continued to take oral BTKi as maintenance treatment after the completion of the induction therapy (median duration of maintenance treatment: 2–18 months). Clinical efficacy was evaluated using the Revised International Working Group Criteria for malignant lymphomas (the Lugano classification). The adverse events were evaluated using CTCAE 5.0 (Common Terminology Criteria for Adverse Events Version 5.0). Tumour burden was assessed based on the sum of the products of diameters (SPD), i.e. the sum of the longest diameter × the diameters perpendicular to the longest diameter of all target lesions, and the treatment effect was determined by assessing changes from baseline during the treatment process. The median follow‐up time in this study was 14.3 months (3–27.5 months).

### 2.3 Statistical analysis

GraphPad Prism 8.3.0 and SPSS 26.0 statistical software were used for statistical processing, analysis and graphing. Initially, normality testing was conducted on the measurement data. Data following a normal distribution (*p* > 0.05) were presented as (x ± s), and the *t*‐test was used for comparison between groups; non‐normally distributed data (*p* < 0.05) were represented as the median (minimum‐maximum) and non‐parametric tests were used for comparison between groups. Enumeration data were expressed as an absolute count and percentage [*n* (%)], and Fisher's exact test was used to analyse intergroup variables. Survival curves were drawn using the Kaplan–Meier method, and the progression‐free survival rate was analysed using the log‐rank test. All statistical comparisons were two‐sided tests, and *p* < 0.05 indicated a statistically significant difference.

## 3 RESULTS

### 3.1 Clinical case characteristics

Among the 23 patients with newly diagnosed DLBCL, the median age was 56 (32–80) years, and nine (39.1%) patients were ≥60 years. The male to female ratio was 15:8. Nineteen (82.6%) patients had stage III‐IV disease, and 16 (69.6%) had extranodal involvement, among whom seven (30.4%) had extranodal involvement ≥2, 13 (56.5%) patients had IPI scores ≥3. Due to the limitations of retrospective research, the RNA of the original tumour tissue was not preserved, so we failed to analyse the cell origin more accurately. According to Han's criteria, eight (34.8%) patients had GCB‐subtype DLBCL, and 15 (65.2%) had non‐GCB‐subtype DLBCL. According to the LymphPlex classification method, 18 had MCD‐like‐subtype DLBCL and five had non‐MCD‐like‐subtype DLBCL (including 2 cases of ST2‐like subtype, 2 cases of BN2‐like subtype, and 1 case of TP53mut subtype). Thirteen (56.5%) patients had double‐expressing DLBCL. Twelve patients received orelabrutinib and 11 received zanubrutinib. Eighteen patients received a BTKi combined with the R‐CHOP regimen and five elderly patients (one patient was 71 years of age and four patients were 80 years of age) received a BTKi combined with the R^2^ regimen. Seventeen patients with newly diagnosed MYD88^mut^ and/or CD79B^mut^ DLBCL who received the standard therapy (R‐CHOP) comprised the control group. Baseline characteristics of age, sex, Ann Arbor stage, extranodal involvement, IPI score, ECOG score, cell of origin, MCD‐like molecular phenotype and double expression were similar between the BTKi + R‐CHOP and R‐CHOP groups (*p* > 0.05) (Table 1).

**TABLE 1:** Patient baseline and clinical characteristics.

	No. (%)			*p*‐value
	BTKi + R‐CHOP	R‐CHOP	BTKi + *R* ^2^	
Characteristic	(*N* = 18)	(*N* = 17)	(*N* = 5)	BTKi + R‐CHOP vs. R‐CHOP
Age				
Age, years	53.5 ± 10.6	52.9 ± 9.7	80 (71–80)	0.859
<60	14 (77.8)	12 (70.6)	0 (0)	0.711
≥60	4 (22.2)	5 (29.4)	5 (100.0)	
Sex				
Female	6 (33.3)	6 (35.3)	2 (40.0)	1
Male	12 (66.7)	11 (64.7)	3 (60.0)	
Ann Arbor stage				
I‐II	4 (22.2)	4 (23.5)	0 (0)	1
III‐IV	14 (77.8)	13 (76.5)	5 (100.0)	
No. of extranodal sites				
0	7 (38.9)	6 (35.3)	0 (0)	0.888
1	6 (33.3)	7 (41.2)	3 (60.0)	
≥2	5 (27.8)	4 (23.5)	2 (40.0)	
IPI				
0–2	10 (55.6)	8 (47.1)	0 (0)	0.740
3–5	8 (44.4)	9 (52.9)	5 (100.0)	
ECOG				
0	10 (55.6)	7 (41.2)	0 (0)	0.678
1	6 (33.3)	7 (41.2)	4 (80.0)	
2	2 (11.1)	3 (17.6)	1 (20.0)	
Cell of origin				
GCB	7 (38.9)	4 (23.5)	1 (20.0)	0.471
Non‐GCB	11 (61.1)	13 (76.5)	4 (80.0)	
HiSeq deep sequencing				
MCD like	14 (77.8)	10 (58.8)	4 (80.0)	0.289
Non‐MCD like	4 (22.2)	7 (41.2)	1 (20.0)	
Double expression				
Yes	10 (55.6)	9 (52.9)	3 (60.0)	1
No	8 (44.4)	8 (47.1)	2 (40.0)	
BTK inhibitor				
Orelabrutinib	10 (55.6)	‐	2 (40.0)	‐
Zanubrutinib	8 (44.4)	‐	3 (60.0)	

*Note*: Unless otherwise noted, data are *N* (%). Among the 23 patients treated with a BTKi, there were 16 cases of MYD88 mutation, including 12 cases of L265P mutation and four cases of other mutations (two cases of S219C mutation and two cases of M232T mutation).

Abbreviations: BTKi, Bruton's tyrosine kinase inhibitor; ECOG, Eastern Cooperative Oncology Group.

### 3.2 Efficacy

**FIGURE 1:**
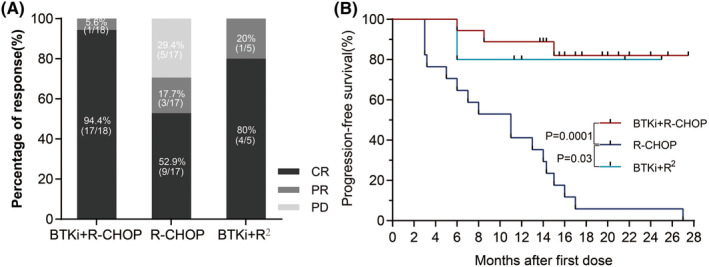
(A) Overall response rates (ORRs) and CR rates for three treatment groups. BTKi + R‐CHOP improved the ORR and CRR versus R‐CHOP (ORR: 100% vs. 70.6%, *p* = 0.019; CRR: 94.4% vs. 52.9%, *p* = 0.007); there was no significant difference among the BTKi+R^2^ and R‐CHOP groups (ORR: 100% vs. 80%, *P* = 0.29). (B) Progression‐free survival in three treatment groups (BTKi + R‐CHOP vs. R‐CHOP, *p* = 0.0001; R‐CHOP vs. BTKi+R^2^, *p* = 0.03). BTKi, Bruton's tyrosine kinase inhibitor.

**FIGURE 2:**
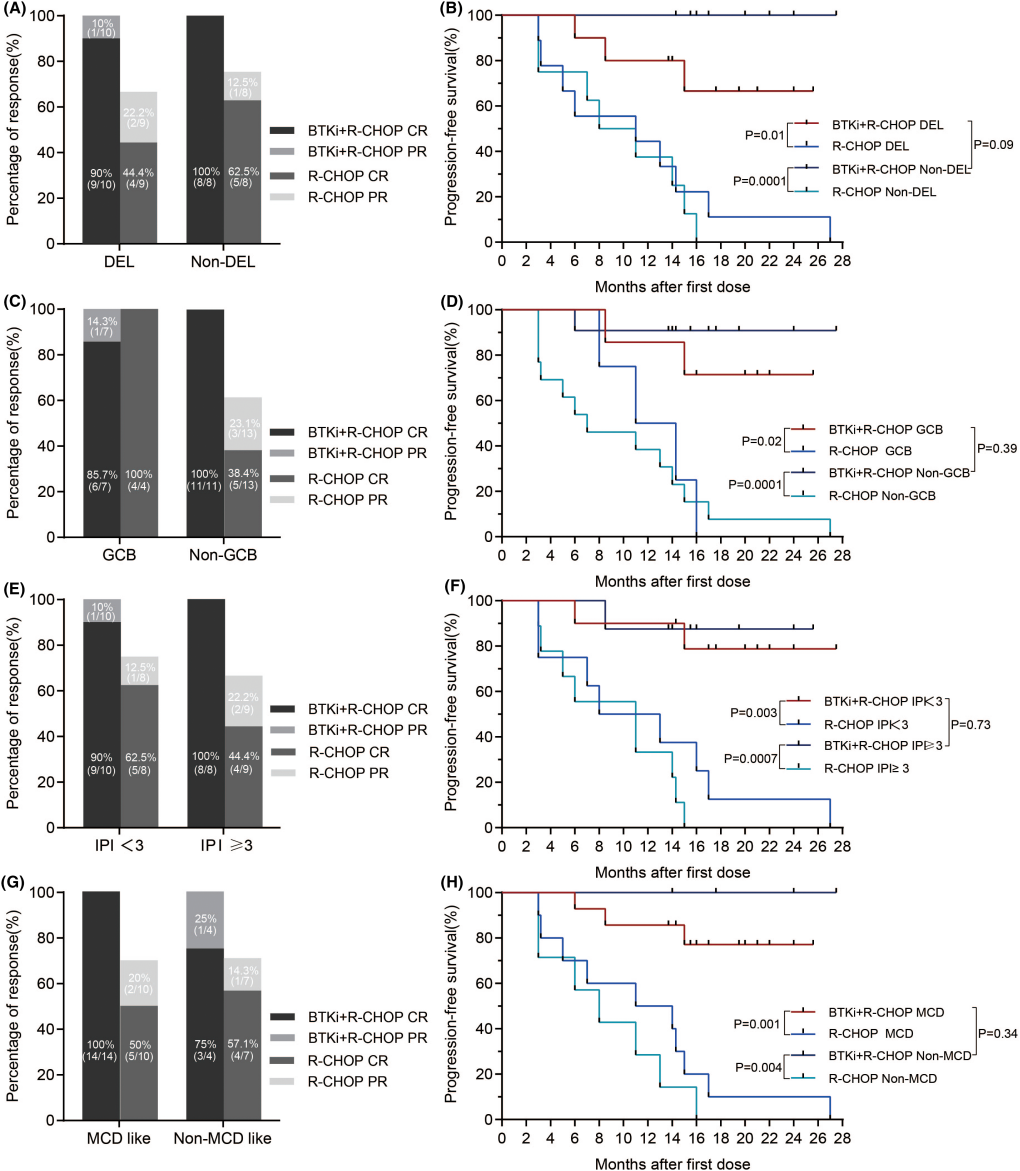
Statistical analysis of overall response rates (ORRs), CRRs and PFS in each subgroup between BTKi + R‐CHOP and R‐CHOP treatment groups. (A) ORRs in the DEL (*p* = 0.087) and non‐DEL (*p* = 0.467) groups. CRRs in the DEL (*p* = 0.057) and non‐DEL (*p* = 0.2) groups. (B) PFS in the DEL (*p* = 0.01) and non‐DEL (*p* = 0.0001) groups. (C) ORRs in the GCB (*p* = 1) and non‐GCB (*p* = 0.041) groups. CRRs in the GCB (*p* = 1) and non‐GCB (*p* = 0.002) groups. (D) PFS in the GCB (*p* = 0.02) and non‐GCB (*p* = 0.0001) groups. (E) ORRs in the IPI <3 (*p* = 0.183) and IPI ≥3 (*p* = 0.206) groups. CRRs in the IPI <3 (*p* = 0.275) and IPI ≥3 (*p* = 0.029) groups. (F) PFS in the IPI <3 (*p* = 0.003) and IPI ≥3 (*p* = 0.0007) groups. (G) ORRs in the MCD‐like subtype (*p* = 0.059) and non‐MCD‐like subtype (*p* = 0.491) groups. CRRs in the MCD‐like subtype (*p* = 0.006) and non‐MCD‐like subtype (*p* = 1) groups. (H) PFS in the MCD‐like subtype (*p* = 0.001) and non‐MCD‐like subtype (*p* = 0.004) groups. BTKi, Bruton's tyrosine kinase inhibitor.

The best ORR, CRR and 1‐year PFS for the 18 BTKi + R‐CHOP patients were 100%, 94.4% and 88.9%, respectively, and were significantly higher than those for the control group (the best ORR, CRR and 1‐year PFS for the 17 R‐CHOP patients were 70.6%, 52.9% and 41.2%, respectively; the *p*‐values were 0.019, 0.007 and 0.0001, respectively). The best ORR, CRR and 1‐year PFS for the five BTKi + *R* ^2^ patients were 100%, 80% and 80% (Figure 1). Figure S1 shows the maximum percent change, compared with baseline, in the SPD of target lesions for the best overall response among the patients with BTKi (orelabrutinib or zanubrutinib) therapy. For the BTKi + R‐CHOP and R‐CHOP treatment groups, a subgroup analysis of 35 patients was performed. A higher ORR in the non‐GCB DLBCL group (100% vs. 61.5%, *p* = 0.04) and a higher CRR in the non‐GCB (100% vs. 38.4%, *p* = 0.002), MCD‐like (100% vs. 50%, *p* = 0.006) and high IPI score (≥3, 100% vs. 44.4%, *p* = 0.03) DLBCL groups were observed in the BTKi + R‐CHOP group than in the control group. For all subtypes, including GCB, non‐GCB, MCD‐like, non MCD‐like, DEL, non‐DEL, and IPI score ≥3 or <3, patients in the BTKi + R‐CHOP group had better PFS than patients in control group (Figure 2).

For the BTKi + R‐CHOP treatment, a subgroup analysis of 18 patients was performed. In terms of cell of origin (GCB vs. non‐GCB, ORR 100% vs. 100%, CRR 85.7% vs. 100%, 1‐year PFS 85.7% vs. 90.9%), dual expression (dual vs. nondual expression, ORR 100% vs. 100%, CR 90% vs. 100%, 1‐year PFS 80% vs. 100%), IPI score (<3 vs. ≥3, ORR 100% vs. 100%, CRR 90% vs. 100%, 1‐year PFS 90% vs. 87.5%) and BTKi type (orelabrutinib vs. zanubrutinib, ORR 100% vs. 100%, CRR 100% vs. 87.5%, 1‐year PFS 100% vs. 75%), there were no significant differences between the subgroups, while BTKi‐type subgroups exhibited statistically significant differences in 1‐year PFS (*p* = 0.028) (Figure 2 and Figure S2).

### 3.3 Adverse events

In all 23 patients treated with a BTKi (orelabrutinib or zanubrutinib), the incidences of any grade and grade 3–4 AEs were 95.7% (22/23) and 65.2% (15/23), respectively. In the BTKi + R‐CHOP treatment group, the incidences of any grade and Grade 3–4 haematological toxicity were 94.4% (17/18) and 66.7% (12/18), respectively, including Grade 3–4 anaemia (nine cases [50%]), neutropenia (eight cases [44.4%]), thrombocytopenia (seven cases [38.9%]) and febrile neutropenia (3 cases [16.7%]). The incidences of any grade and Grade 3–4 non‐haematological toxicity were 61.1% (11/18) and 27.8% (5/18), respectively. The most common Grade 3–4 non‐haematological toxicity was infection (5 cases, 27.8%, of which 4 were pulmonary infections and 1 was sepsis). In the R‐CHOP treatment group, the incidences of any grade and Grade 3–4 haematological toxicity were 100% (17/17) and 70.6% (12/17), respectively. The incidences of any grade and Grade 3–4 non‐haematological toxicity were 52.9% (9/17) and 41.2% (7/17), respectively. The most common Grade 3–4 non‐haematological toxicity was infection (6/17, 35.3%). There were no significant differences in Grade 3–4 haematological toxicity (*p* = 1) and Grade 3–4 non‐haematological toxicity (*p* = 0.49) between the BTKi+R‐CHOP treatment group and R‐CHOP treatment group (Table 2).

**TABLE 2:** Treatment‐emergent AEs.

	No. (%)					
	BTKi + R‐CHOP (*N* = 18)		R‐CHOP (*N* = 17)		BTKi + *R* ^2^ (*N* = 5)	
	TEAEs	Grade ≥3 TEAEs	TEAEs	Grade ≥3 TEAEs	TEAEs	Grade ≥3 TEAEs
Haematological toxicity						
Neutropenia	12 (66.7)	8 (44.4)	15 (88.2)	7 (41.2)	4 (80)	2 (40)
Anaemia	16 (88.9)	9 (50)	16 (94.1)	8 (47.1)	5 (100)	0 (0)
Thrombocytopenia	13 (72.2)	7 (38.9)	14 (82.4)	7 (41.2)	4 (80)	0 (0)
Febrile neutropenia	3 (16.7)	3 (16.7)	4 (23.5)	4 (23.5)	0 (0)	0 (0)
Non‐haematological toxicity						
Pneumonia	4 (22.2)	4 (22.2)	6 (35.3)	6 (35.3)	1 (20)	1 (20)
Urinary tract infection	0 (0)	0 (0)	0 (0)	0 (0)	1 (20)	0 (0)
Sepsis	1 (5.6)	1 (5.6)	2 (11.8)	2 (11.8)	0 (0)	0 (0)
Diarrhoea	5 (27.8)	0 (0)	2 (11.8)	1 (5.9)	0 (0)	0 (0)
Aminotransferase increased	7 (38.9)	0 (0)	10 (58.8)	1 (5.9)	1 (20)	1 (20)
Ecchymosis	0 (0)	0 (0)	0 (0)	0 (0)	1 (20)	0 (0)
Arrhythmia	3 (16.7)	0 (0)	2 (11.8)	0 (0)	0 (0)	0 (0)

*Note*: All data are *N* (%).

Abbreviation: BTKi, Bruton's tyrosine kinase inhibitor; TEAEs, treatment emergent adverse events.

Among the five patients in the BTKi + R^2^ treatment group, 2 (2/5,40%) had Grade ≥3 haematological toxicity, both with neutropenia. Two patients (2/5) had grade ≥3 non‐haematological toxicity (pulmonary infection and elevated transaminases) (Table 2). All patients had no Grade 3 or higher bleeding or cardiovascular events. A patient temporarily discontinued BTKi for a week due to rash on the extremities. After the rash resolved, the patient continued the use of BTKi and did not have rash again. The median recovery time was 9 days from Grade 3 haematological toxicity and 7 days from Grade 3 non‐haematological toxicity (pulmonary infection).

## 4 DISCUSSION

In this study, 40 newly diagnosed DLBCL patients with MYD88 and/or CD79B mutations had a high extranodal involvement rate (67.5%) and high IPI score (≥3 55%), findings that are consistent with previous reports on this type of lymphoma.^3^ The efficacy of zanubrutinib or orelabrutinib combined with R‐CHOP in 18 patients was similar to that of ibrutinib combined with R‐CHOP in young (<60 years) MCD DLBCL patients (1‐year EFS, 100%**)** in terms of the ORR (100%), CR rate (94.4%) and 1‐year PFS (88.9%), and both were significantly better than R‐CHOP alone according to the treatment of 17 patients with this type of DLBCL who were treated with R‐CHOP alone in this study (ORR 70.6%, CR rate 52.9% and 1‐year PFS 41.2%). Moreover, when evaluating efficacy, it is necessary to fully consider that 14 patients in the BTKi+R‐CHOP group received a BTKi as maintenance therapy, which was different from precious reports.^13^

In the era of immunochemotherapy, non‐GCB DLBCL, a high IPI score and MCD‐like subtype are all predictors of a poor prognosis for patients with DLBCL.^23^, ^24^, ^25^, ^26^, ^27^ Our study suggests that a therapeutic regimen containing BTKis, compared with the standard regimen, can significantly improve the CRR in DLBCL patients with the above high‐risk factors. A subgroup analysis of 18 patients in the BTKi + R‐CHOP treatment group suggested that there were no statistically significant differences in ORR, CRR and PFS between GCB versus non‐GCB DLBCL, non‐DEL versus DEL or IPI ≥3 versus <3. Therefore, the addition of a BTKi may improve the prognosis of non‐GCB, DEL or high‐IPI‐score DLBCL patients with MYD88^mut^ and/or CD79B^mut^, which is similar to findings of previous studies.^12^, ^28^ Although the zanubrutinib and orelabrutinib subgroups had significant difference in 1‐year PFS (*p* = 0.028), which of the two drugs has better efficacy for DLBCL needs to be verified by expanding the sample size.

The most used treatment regimen in elderly patients with DLBCL is R‐miniCHOP. According to multiple studies, the regimen has an ORR of 73%, a CRR of 53%–62%, and 1‐year PFS close to 60%.^29^, ^30^ Elderly DLBCL patients with MYD88 and/or CD79B mutations have a poorer prognosis and the expected efficacy is worse. In our study, five elderly DLBCL patients with MYD88 and/or CD79B mutations were treated with the BTKi (orelabrutinib or zanubrutinib) + *R* ^2^ regimen and achieved an ORR of 100%, a CRR of 80%, and 1‐year PFS of 80%, which are significantly better than the historical data for R‐miniCHOP treatment and Ibrutinib + *R* ^2^ treatment (ORR 42.9% CRR 28.6% for MYD88^L265P^CD79B^mut^ and MYD88^wt^CD79B^mut^ DLBCL).^31^ In the past, for MYD88^L265P^ DLBCL without CD79B mutation, the benefit of BTKis has been controversial.^12^, ^32^ In this study, two patients with the non‐MCD subtype MYD88^L265P^CD79B^wt^ DLBCL both achieved a CR and sustained remission up to 13.7 months. However, due to the small number of cases, the efficacy and benefit of BTKis for this type of DLBCL need to be further analysed using a larger sample size.

Results from the PHOENIX study indicated that DLBCL patients ≥60 years did not benefit from ibrutinib combined with R‐CHOP due to poor safety.^13^ Orelabrutinib and zanubrutinib are kinase‐specific BTKis with better safety profiles^33^, ^34^ and better synergize with CD20 monoclonal antibodies.^35^ In this study, the combination of orelabrutinib and zanubrutinib with R‐CHOP was well tolerated, and compared with R‐CHOP, there were no significant differences in Grade 3–4 haematological toxicity (*p* = 1) and Grade 3–4 non‐haematological toxicity (*p* = 0.49). The incidences of AEs of any grade (94.4% vs. 99%) or ≥Grade 3 (66.7% vs. 87.1%) were also similar in those reported in a previous study, with no additional toxicity identified.^13^ More interestingly, the BTKi + *R* ^2^ regimen also showed a good safety profile in five elderly patients; in particular, compared with the R‐miniCHOP regimen, ≥Grade 3 AEs (53%)^30^ appeared to be reduced with the BTKi + *R* ^2^ regimen (40%). However, the number of cases in this study was small, and thus, compared with R‐miniCHOP regimen, a more definite conclusion regarding safety needs to be reached by further head‐to‐head clinical trials with an expanded sample size. The small sample size of this study might result in clinical bias in the safety data, and more accurate safety data will need confirmation from further clinical trials.

This study suggested that the addition of zanubrutinib or orelabrutinib in first‐line treatment achieved good safety and efficacy in 23 newly diagnosed DLBCL patients with combined MYD88^mut^ and/or CD79B^mut^.

## AUTHOR CONTRIBUTIONS

**Ting Deng:** Data curation (lead); formal analysis (lead); investigation (lead); methodology (lead); software (lead); writing – original draft (lead). **Shiyuan Zhang:** Data curation (equal); investigation (equal); software (equal); writing – original draft (equal). **Min Xiao:** Data curation (equal); methodology (equal); writing – review and editing (equal). **Jia Gu:** Data curation (equal); methodology (equal); software (equal); writing – original draft (equal). **Liang Huang:** Conceptualization (equal); methodology (equal); supervision (equal); writing – review and editing (equal). **Xiaoxi Zhou:** Conceptualization (lead); methodology (lead); project administration (lead); supervision (lead); writing – review and editing (lead).

## FUNDING INFORMATION

This study was supported by the National Natural Science Foundation of China (Grant No.82170167).

## CONFLICT OF INTEREST STATEMENT

The authors declare that they have no known competing financial interests or personal relationships that could have appeared to influence the work reported in this paper.

## ETHICS STATEMENT

This study was approved by the Medical Ethics Committee of Tongji Hospital, Tongji Medical College of Huazhong University of Science and Technology, in accordance with the Declaration of Helsinki. The patients in this study gave their written informed consent in accordance with the Declaration of Helsinki.

## Supporting information


Figure S1.

Figure S2.

Table S1.

## Data Availability

The dataset(s) supporting the findings of this study are included within the article.

## References

[1] Schmitz R , Wright GW , Huang DW , et al. Genetics and pathogenesis of diffuse large B‐cell lymphoma. N Engl J Med. 2018;378(15):1396‐1407. 29641966 10.1056/NEJMoa1801445 PMC6010183

[2] Lacy SE , Barrans SL , Beer PA , et al. Targeted sequencing in DLBCL, molecular subtypes, and outcomes: a haematological malignancy research network report. Blood. 2020;135(20):1759‐1771. 32187361 10.1182/blood.2019003535 PMC7259825

[3] Lee JH , Jeong H , Choi JW , Oh HE , Kim YS . Clinicopathologic significance of MYD88 L265P mutation in diffuse large B‐cell lymphoma: a meta‐analysis. Sci Rep. 2017;7(1):1785. 28496180 10.1038/s41598-017-01998-5 PMC5431939

[4] Dubois S , Viailly PJ , Bohers E , et al. Biological and clinical relevance of associated genomic alterations in MYD88 L265P and non‐L265P‐mutated diffuse large B‐cell lymphoma: analysis of 361 cases. Clin Cancer Res. 2017;23(9):2232‐2244. 27923841 10.1158/1078-0432.CCR-16-1922

[5] Reddy A , Zhang J , Davis NS , et al. Genetic and functional drivers of diffuse large B cell lymphoma. Cell. 2017;171(2):481‐494. 28985567 10.1016/j.cell.2017.09.027 PMC5659841

[6] Vermaat JS , Somers SF , de Wreede LC , et al. MYD88 mutations identify a molecular subgroup of diffuse large B‐cell lymphoma with an unfavorable prognosis. Haematologica. 2020;105(2):424‐434. 31123031 10.3324/haematol.2018.214122 PMC7012469

[7] Rovira J , Karube K , Valera A , et al. MYD88 L265P mutations, but No other variants, identify a subpopulation of DLBCL patients of activated B‐cell origin, extranodal involvement, and poor outcome. Clin Cancer Res. 2016;22(11):2755‐2764. 26792260 10.1158/1078-0432.CCR-15-1525

[8] Phelan JD , Young RM , Webster DE , et al. A multiprotein supercomplex controlling oncogenic signalling in lymphoma. Nature. 2018;560(7718):387‐391. 29925955 10.1038/s41586-018-0290-0 PMC6201842

[9] Yang Y , Shaffer AL , Emre NC , et al. Exploiting synthetic lethality for the therapy of ABC diffuse large B cell lymphoma. Cancer Cell. 2012;21(6):723‐737. 22698399 10.1016/j.ccr.2012.05.024 PMC4059833

[10] Davis RE , Ngo VN , Lenz G , et al. Chronic active B‐cell‐receptor signalling in diffuse large B‐cell lymphoma. Nature. 2010;463(7277):88‐92. 20054396 10.1038/nature08638 PMC2845535

[11] Yang HY , Xiang B , Song YQ , et al. Zanubrutinib monotherapy for relapsed or refractory non‐germinal center diffuse large B‐cell lymphoma. Blood Adv. 2022;6(6):1629‐1636. 34638136 10.1182/bloodadvances.2020003698 PMC8941452

[12] Wilson WH , Young RM , Schmitz R , et al. Targeting B cell receptor signaling with ibrutinib in diffuse large B cell lymphoma. Nat Med. 2015;21(8):922‐926. 26193343 10.1038/nm.3884 PMC8372245

[13] Younes A , Sehn LH , Johnson P , et al. Randomized phase III trial of ibrutinib and rituximab plus cyclophosphamide, doxorubicin, vincristine, and prednisone in non‐germinal center B‐cell diffuse large B‐cell lymphoma. J Clin Oncol. 2019;37(15):1285‐1295. 30901302 10.1200/JCO.18.02403 PMC6553835

[14] Wilson WH , Wright GW , Huang DW , et al. Effect of ibrutinib with R‐CHOP chemotherapy in genetic subtypes of DLBCL. Cancer Cell. 2021;39(12):1643‐1653. 34739844 10.1016/j.ccell.2021.10.006 PMC8722194

[15] Armitage JO , Potter JF . Aggressive chemotherapy for diffuse histiocytic lymphoma in the elderly: increased complications with advancing age. J Am Geriatr Soc. 1984;32(4):269‐273. 6368652 10.1111/j.1532-5415.1984.tb02020.x

[16] Westin JR , Davis RE , Feng L , et al. Smart start: rituximab, lenalidomide, and ibrutinib in patients with newly diagnosed large B‐cell lymphoma. J Clin Oncol. 2023;41(4):745‐755. 35952327 10.1200/JCO.22.00597 PMC10489211

[17] Zhu J , Cheng Y , Guo J , et al. The Chinese Society of Clinical Oncology (CSCO) diagnosis and treatment guidelines for malignant lymphoma 2023. Guidelines Working Committee of Chinese Society of Clinical Oncology. Vol 30. People's Medical Publishing House; 2023.

[18] Swerdlow SH , Campo E , Pileri SA , et al. The 2016 revision of the World Health Organization classification of lymphoid neoplasms. Blood. 2016;127(20):2375‐2390. 26980727 10.1182/blood-2016-01-643569 PMC4874220

[19] Hans CP , Weisenburger DD , Greiner TC , et al. Confirmation of the molecular classification of diffuse large B‐cell lymphoma by immunohistochemistry using a tissue microarray. Blood. 2004;103(1):275‐282. 14504078 10.1182/blood-2003-05-1545

[20] Hu S , Xu‐Monette ZY , Tzankov A , et al. MYC/BCL2 protein coexpression contributes to the inferior survival of activated B‐cell subtype of diffuse large B‐cell lymphoma and demonstrates high‐risk gene expression signatures: a report from the international DLBCL rituximab‐CHOP consortium program. Blood. 2013;121(20):4021‐4031. 23449635 10.1182/blood-2012-10-460063 PMC3709650

[21] The International Non‐Hodgkin's Lymphoma Prognostic Factors . A predictive model for aggressive non‐Hodgkin's lymphoma. N Engl J Med. 1993;329(14):987‐994. 8141877 10.1056/NEJM199309303291402

[22] Oken MM , Creech RH , Tormey DC , et al. Toxicity and response criteria of the eastern cooperative oncology group. Am J Clin Oncol. 1982;5(6):649‐655. 7165009

[23] Shen R , Fu D , Dong L , et al. Simplified algorithm for genetic subtyping in diffuse large B‐cell lymphoma. Signal Transduct Target Ther. 2023;8(1):145. 37032379 10.1038/s41392-023-01358-y PMC10083170

[24] Scott DW , Mottok A , Ennishi D , et al. Prognostic significance of diffuse large B‐cell lymphoma cell of origin determined by digital gene expression in formalin‐fixed paraffin‐embedded tissue biopsies. J Clin Oncol. 2015;33(26):2848‐2856. 26240231 10.1200/JCO.2014.60.2383 PMC4554747

[25] Rosenwald A , Wright G , Chan WC , et al. The use of molecular profiling to predict survival after chemotherapy for diffuse large‐B‐cell lymphoma. N Engl J Med. 2002;346:1937‐1947. 12075054 10.1056/NEJMoa012914

[26] Ruppert AS , Dixon JG , Salles G , et al. International prognostic indices in diffuse large B‐cell lymphoma: a comparison of IPI, R‐IPI, and NCCN‐IPI. Blood. 2020;135(23):2041‐2048. 32232482 10.1182/blood.2019002729

[27] Ziepert M , Hasenclever D , Kuhnt E , et al. Standard international prognostic index remains a valid predictor of outcome for patients with aggressive CD20+ B‐cell lymphoma in the rituximab era. J Clin Oncol. 2010;28(14):2373‐2380. 20385988 10.1200/JCO.2009.26.2493

[28] Johnson P , Balasubramanian S , Hodkinson B , et al. Clinical impact of ibrutinib with R‐CHOP in untreated non‐GCB DLBCL Co‐expressing BCL2 and MYC genes in the phase 3 Phoenix trial. Blood. 2019;134(S1):354. 10.1182/bloodadvances.2022009389 PMC10188634 36696540

[29] Peyrade F , Jardin F , Thieblemont C , et al. Attenuated immunochemotherapy regimen (R‐miniCHOP) in elderly patients older than 80 years with diffuse large B‐cell lymphoma: a multicentre, single‐arm, phase 2 trial. Lancet Oncol. 2011;12(5):460‐468. 21482186 10.1016/S1470-2045(11)70069-9

[30] Oberic L , Peyrade F , Puyade M , et al. Subcutaneous rituximab‐MiniCHOP compared with subcutaneous rituximab‐MiniCHOP plus lenalidomide in diffuse large B‐cell lymphoma for patients age 80 years or older. J Clin Oncol. 2021;39(11):1203‐1213. 33444079 10.1200/JCO.20.02666

[31] Xu PP , Shi ZY , Qian Y , et al. Ibrutinib, rituximab, and lenalidomide in unfit or frail patients aged 75 years or older with de novo diffuse large B‐cell lymphoma: a phase 2, single‐arm study. Lancet Healthy Longev. 2022;3(7):e481‐e490. 36102758 10.1016/S2666-7568(22)00123-4

[32] Jiang S , Qin Y , Gui L , et al. Genomic alterations and MYD88MUT variant mapping in patients with diffuse large B‐cell lymphoma and response to ibrutinib. Target Oncol. 2020;15(2):221‐230. 32239385 10.1007/s11523-020-00710-4

[33] Hillmen P , Brown JR , Eichhorst BF , et al. ALPINE: zanubrutinib versus ibrutinib in relapsed/refractory chronic lymphocytic leukemia/small lymphocytic lymphoma. Future Oncol. 2020;16(10):517‐523. 32207333 10.2217/fon-2019-0844

[34] Tam CS , Opat S , D'Sa S , et al. A randomized phase 3 trial of zanubrutinib vs ibrutinib in symptomatic Waldenström macroglobulinemia: the ASPEN study. Blood. 2020;136(18):2038‐2050. 32731259 10.1182/blood.2020006844 PMC7596850

[35] Yu H , Wang X , Li J , et al. Addition of BTK inhibitor orelabrutinib to rituximab improved anti‐tumor effects in B cell lymphoma. Mol Ther Oncolytics. 2021;21:158‐170. 33981831 10.1016/j.omto.2021.03.015 PMC8082047

