# Supplementary figures and images for: A single‐centre, real‐world study of BTK inhibitors for the initial treatment of MYD88mut /CD79Bmut diffuse large B‐cell lymphoma
Source: Cancer Med. 2024 Mar 8;13(4):e7005. doi: 10.1002/cam4.7005 (PMC10923040; doi:10.1002/cam4.7005)

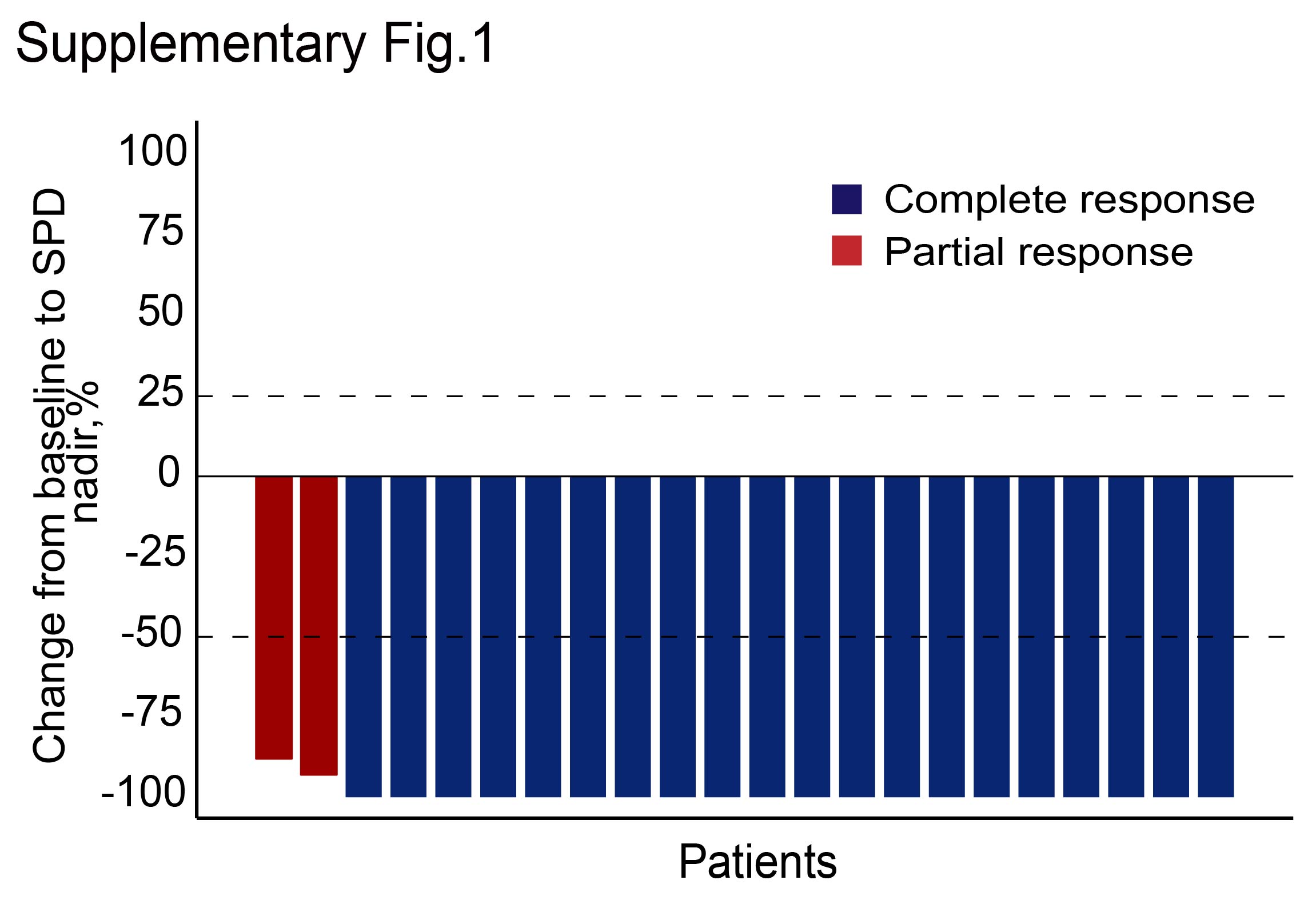

Supplement: Supplementary file 1 — Figure S1. [file CAM4-13-e7005-s002.jpg]

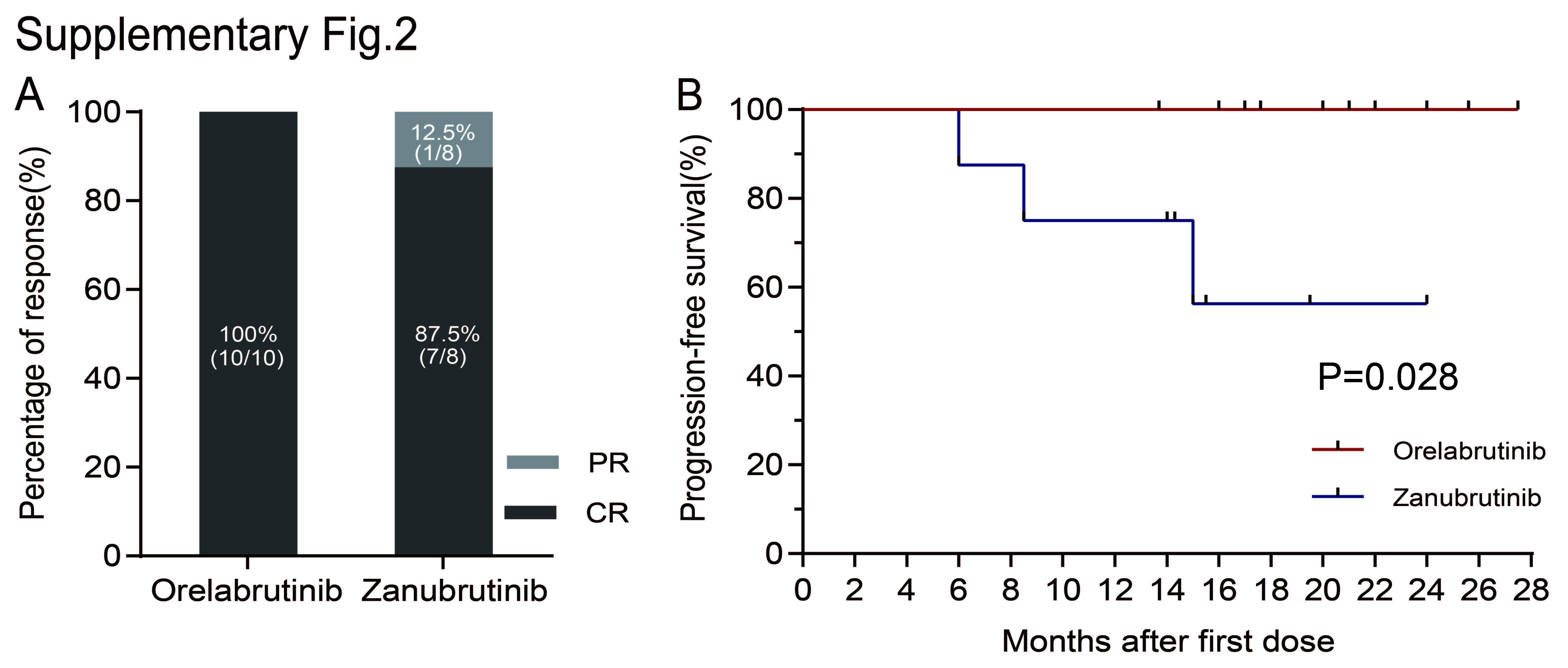

Supplement: Supplementary file 2 — Figure S2. [file CAM4-13-e7005-s001.jpg]
