# Supplementary material for: A single‐centre, real‐world study of BTK inhibitors for the initial treatment of MYD88mut /CD79Bmut diffuse large B‐cell lymphoma
Source: Cancer Med. 2024 Mar 8;13(4):e7005. doi: 10.1002/cam4.7005 (PMC10923040; doi:10.1002/cam4.7005)
Supplement: Supplementary file 3 — Table S1. [file CAM4-13-e7005-s003.docx]

**A single-centre, real-world study of BTK inhibitors for the initial treatment of MYD88^mut^/CD79B^mut^ diffuse large B-cell** **lymphoma**

Ting Deng ^1,b^, Shiyuan Zhang^a^, Min Xiao^a^, Jia Gu^a^, Liang Huang^a^, Xiaoxi Zhou^* , a^

a. Department of Hematology, Tongji Hospital, Tongji Medical College, Huazhong University of Science and Technology, Wuhan, Hubei 430030, PR China

b. Department of Hematology, Chongqing Fifth People's Hospital, Chongqing, Chongqing 400062, PR China

* Corresponding authors: Xiaoxi Zhou, Tel: +86-27- 83665007; Fax: +86-27-83662680, E-mail: cello316@163.com

**Supplementary Data**

**Supplementary Table 1**

**Supplementary Table 1**

| **ID** | **Cell of origin** | **Immunohistochemical** | | **Sex** | **Age** | **Ann Arbor stage** | **IPI** | **ECOG** | **FISH (Rearran-gements in BCL2, BCL6, and MYC)** | | **NGS** | **Extra-nodal sites** | **Chemoth-erapy regimens** | | **BTKi** | | **BTKi** **mainten-ance time（Month）** | |
| --- | --- | --- | --- | --- | --- | --- | --- | --- | --- | --- | --- | --- | --- | --- | --- | --- | --- | --- |
| 1 | non-GCB | | CD20(+), CD3(-), CD30(scatteredly +), CD5(-), CD56(-), Ki67(+, LI 90%), TIA-1(-), CD19(+), pax-5(+), MyoD1, Myogenin(-), Mum-1(+), Bcl-2(+), Bcl-6(+), C-myc(+, 20%), Cyclin-D1(-), CD10(-). | M | 37 | III | 2 | 0 | Negative | | CD79B  EP300  PIM1  TET2 | - | 6R-CHOP | | Orelabrutinib | | 0 | |
| 2 | GCB | | CD20(L26)(+), CD20(+), PAX5(SP34)(+), CD79a(weak+), CD10( +), BCL2(SP66)(+), BCL6( 60%+), C-MYC( 30%+), CD19(+), CD22(weak+), LEF1(partially+), MUM1( +), MNDA(scatteredly +), CD21( weak+), FDC networkCD23, CD35(-), TIA1(partially+), CD43(partially weak+), CD30(VENTANA)(-), CD30(+), HGAL(-), CyclinD1(-), SOX11(-), IgD(-)PCK(-), Ki-67( LI: approximately 90%); background cells CD3(2GV6)(+), CD4(+), CD5(+), CD8(+), GrB(-). | F | 56 | IV | 3 | 1 | Fusion of BCL6 | | CD79B  B2M  BTG1  BTG2  DTX1  TET2 | Pancreas  duode-num | 6R-CHOP | | Orelabrutinib | | 17 | |
| 3 | non-GCB | | CD20(+), CD19(+), CD22(+)PAX5(+), MUM1(+), BCL6(+), C-MYC(40%+), BCL2(60% +), CD21(+), CD10(-), CD23(-), CD30(-), CD3(-), CD43(-), CD5(-), Ki-67 LI: 90%. | M | 40 | IV | 2 | 0 | Negative | | MYD88 KMT2D  TNFAIP3  STAT6  SOCS1  GNA13  DUSP2  TBL1XR1 | Liver | 6R-CHOP | | Orelabrutinib | | 4 | |
| 4 | GCB | | CD20(+), CD19(+), CD79a(+), CD10(+), BCL2(50%+), BCL6(+), MUM1(+), CD21, CD23 and CD35(residual FDC network+), CD5(-), CyclinD1(-), SOX11(-), IgD(-), C-MYC(60%+), CD30(-), CD3(-), CD43(-), Ki-67(LI: approximately 60%). | M | 51 | II | 0 | 0 | Negative | | MYD88  CD79B | - | 6R-CHOP | | Orelabrutinib | | 4 | |
| 5 | GCB | | CD20(+), CD3(+), CD5(+), CD10(+), Bcl-2(+), Bcl-6(+), Pax-5(+), MUM-1(+), CD21(residual FDC network+), CD30(-), CD15(-), CD79a(+), Ki-67(Li 40%). | F | 66 | II | 2 | 0 | Negative | | MYD88  TNFAIP3  KMT2D  CD79B  CREBBP | - | 6R-CHOP | | Orelabrutinib | | 4 | |
| 6 | non-GCB | | CD20(+), CD20(+), CD19(+), PAX5(+), BCL2(95%+), BCL6(90%+), C-MYC(40%+), MUM1(80%+), CD22(-), CD5(-), CD10(-), CyclinD1(-), CD35(-), CD30(-), CD30(+), CD3(-), CD43(-), PCK(-), P40(-), Ki-67 LI: approximately 90%. | M | 55 | II | 0 | 0 | Negative | | KMT2D  CD79B  BTG1  PIM1  DUSP2 | - | 6R-CHOP | | Orelabrutinib | | 12 | |
| 7 | non-GCB | | CD20(+), CD19(+), CD22(+), PAX5(+), BCL2(90%+), BCL6(90%+), C-MYC(40%+), MUM1(95%+), CD3(-), CD43(-), CD5(-), CD10(-), CyclinD1(-), CD35(-), CD30(3%+), Ki-67 LI: approximately 90%. | F | 54 | IV | 3 | 1 | Fusion of BCL6 | | MYD88  CD70  BTG2  IRF4 | Adrenal glands  Uterus  Bone | 6R-CHOP | | Orelabrutinib | | 9 | |
| 8 | GCB | | CD20(+), CD19(+), PAX5(+), CD22(weak+), BCL2(5%+), BCL6(30%+), C-MYC(40%+), CD10(+), MUM1(approximately 40%+), CD30(approximately 1%+), CyclinD1(-), CD23(-), CD35(-), CD3(-), CD56(-), GFAP(weak+), Oligo2(-), SALL4(-), Ki-67 LI: approximately 70%. | M | 58 | IV | 2 | 0 | Negative | | MYD88  CD79B  GNA13  PIM1  BTG2  DTX1  KMT2D | Frontal lobe | 6R-CHOP | | Orelabrutinib | | 0 | |
| 9 | non-GCB | | MPO+ in myelocytes, CD34+ in blood vessels, CD15 slightly + in guanulocytes, CD42b+ in megakaryocytes, atypical lymphocytes, CD3-, CD5-, CD10-, CD19+, CD20+, CD22+, bc1-2 (+) , bc1-6 (+), MUM1+, CD30-, K1-67L1>90% | M | 32 | IV | 2 | 0 | Fusion of MYC | | CD79B  PIM1  BTG1 BTG2  CCND3  DTX1 | Bone marrow | 6R-CHOP | | Orelabrutinib | | 0 | |
| 10 | GCB | | CD20 (+) , CD19 (+), CD22(+) , PA5(weak+) , CD5 (+) , CD10 (+) , BCL2 (95%+) , BCL6 (-) , CyclinD1 (-) , CD35 (-) , C-MYC(1%+) , MUM1(15%+), CD30 (-) , CD3 (-) , CD43 (-), CD56(-) , ALK (-) , Syn (-), S-100 (-) , PCK (-) , EMA (-) , Ki-67LI: approximately 90%. | F | 57 | III | 3 | 2 | Negative | | MYD88  PIM1  BGT1  BGT2  TBL1XR1 | - | 6R-CHOP | | Orelabrutinib | | 0 | |
| 11 | non-GCB | | CD20(+), CD19(+), CD22(+), PAX5(+), CD79a(+), CD5(+), BCL2(80%+), MUM1(+), BCL6(+), C-MYC(40%+), LEF1(partially+), CD10(-), CyclinD1(-), SOX11(-), IgD(-), MNDA(-), CD21, CD23 and CD35(-, indicating a small amount of residual FDC network), CD3(-), CD43(-), PCK(-), CD30(+), Ki-67 LI: approximately 85%. | M | 74 | IV | 4 | 2 | Negative | | MYD88  CD79B  PIM1  CCND3  KMT2D | Liver  Bone | 6R-CHOP | | Zanubrutinib | | 18 | |
| 12 | non-GCB, | | CD20(+), CD19(+), CD22(+), PAX5(+), BCL2(95%+), BCL6(40%+), C-MYC(60%+), MUM1(+), CD43(+), CD21 and CD35(FDC network-), CD10(-), CyclinD1(-), CD3(-), CD5(-), IgD(-), MNDA(-), LEF1(-), κ(slightly +), λ(slightly +), PCK(-), Ki-67(LI: approximately 70%). | F | 55 | IV | 4 | 1 | Negative | | MYD88  CD79B  PIM1  BTG1 | Lung  Nasophar-ynx  Bone marrow | 6R-CHOP | | Zanubrutinib | | 6 | |
| 13 | GCB | | CD20(+), CD19(+), CD22(+), PAX5(+), CD10(+), BCL2(80%+), BCL6(80%+), C-MYC(40%+), MUM1(approximately 60%+), CD3(-), CD43(-), CD5(-), CyclinD1(-), SOX11(-), IgD(-), CD21(-), CD23(-), CD35(-), ALK-1A4(-), CD30(-, positive control+), Ki-67 LI: approximately 80%. | M | 65 | IV | 3 | 1 | Negative | | MYD88  CD79B  IRF4  PIM1  DTX1 | Heart | 6R-CHOP | | Zanubrutinib | | 2.5 | |
| 14 | non-GCB | | CD19+ CD20+ CD79a+ CD21partially+ BCL2+ BCL6(+)MUM-1+ C-MYC(25%+)CD3- CD5- CD23- CyclinD1- CD30- CD10- Ki-67(Li:80%). | M | 46 | IV | 1 | 0 | Negative | | CD79B  DUSP2  ZFP36L1  PIM1  ARID1A  KMT2D | Ileum | 6R-CHOP | | Zanubrutinib | | 6 | |
| 15 | non-GCB | | CK-, CD20+, CD79a+, CD3-, CD5weak+, CD10-, MUM1+, BCL6-, cyclinD1-, CD30-, Ki-67 70%+. | F | 64 | IV | 3 | 1 | Negative | | MYD88  CD79B  PIM1  BTG1  TBL1XR1  ,KMT2D  DUSP2  DTX1 | Kidney | 6R-CHOP | | Zanubrutinib | | 6 | |
| 16 | non-GCB | | CD20(+), CD19(+), CD22(+), PAX5(+), BCL2(100%+), MUM1(+), C-MYC(50%+), BCL6(1%+), CD79b(+), CD10(-), LEF1(+), CyclinD1(-), SOX11(-), IgD(-), CD34(-), TdT(-), CD21, CD23 and CD35(residual FDC network+), CD30(approximately 1%+, positive control+), CD3(-), CD5(-), CD43(-), ALK-1A4(-), Ki-67(LI: approximately 90%). | M | 46 | II | 1 | 0 | Negative | | MYD88  CD79B  PIM1  IRF4 | - | 6R-CHOP | | Zanubrutinib | | 2 | |
| 17 | non-GCB | | CD20(+), CD19(+), BCL2(50%+), MUM1(+), BCL6(80%+), C-MYC(60%+), CyclinD1(5%+), CD35(partially+), CD10(-), CD30(individually +, positive control+), CD3(-), CD5(-), CD4(-), CD8(-), CD56(-), GrB(-), TIA-1(-), PCK(-), Ki-67(LI 80%). | M | 51 | IV | 3 | 0 | Negative | | MYD88  PIM1  BTG2 | Cerebrosp-inal fluid  Lung  Bone marrow | 6R-CHOP | | Zanubrutinib | | 9 | |
| 18 | GCB | | CD3(+in the interfollicular area), CD20(+), CD21(FDC network+), Ki-67(LI approximately 80%), CD30(-), ALK(1A4)(0), CD10(-), CD38(Z+, disappearance of strongly positive cells in the germinal center), Bcl-2(60%+), Bcl-6(+), HGAL(+), C-myc(>40%),MUM1(-). | M | 56 | III | 2 | 1 | Fusion of BCL6 | | CD79B  TP53^mut^  BTG1 | - | 6R-CHOP | | Zanubrutinib | | 10 | |
| 19 | non-GCB | | CD20+, CD19+, CD22(+), CD79a(+), CD79b(weak+), PAX5+, BCL6(approximately 10%weak+), C-MYC(approximately 90%+), MUM1(approximately 95%+), BCL2(60%+) , CD10(-), CD5(-), CyclinD1-, CD21(-), CD23-, CD35-, CD3-, PD1-, CD43-, CD30-, Ki-67 LI: approximately 100%, | F | 71 | IV | 4 | 1 | Negative | MYD88  CD79B  PIM1 TNFA1P3  TBL1XR1  PRDM1  SGK1 | | Heart  Bone | | 6R^2^ | Orelabrutinib | | | 0 |
| 20 | GCB | | CD20(+), CD19(+), PAX-8(+), BCL6(+), CD10(+), MUM1(slightly weak+), C-MYC(approximately 30%+), CD30(-), CD30(positive control)(+), CD5(-), BCL2(-), CyclinD1(-), CD3(-), CD43(-), CD34(-), TdT(-), GATA-3(-), UroplakinII(-), PCK(-), CK8/18(-), CK5/6(-), P40(-), P63(-), CK7(-), CK20(-), CD56(-), Syn(-), CgA(-), PSA(-), Nkx3.1(-), P504S(-), S-100(-), Ki-67LI: approximately 95%. | M | 80 | IV | 5 | 2 | Negative | MYD88  CD79B  PIM1  BTG1  DTX1 | | Scrotum  Ureter | | 6R^2^ | Orelabrutinib | | | 12 |
| 21 | non-GCB | | CD20(+,positive control+), CD19(+), CD22(+), PAX5(+), MUM1(+), CD5(slightly weak+), BCL2(approximately 90% +), BCL6(approximately 20%+), C-MYC(approximately 50%+), CD30(approximately 1%+,positive control+),CD10(-), CyclinD1(-), SOX11(-), IgD(-), CD21(-), CD23(-), CD35(-), LEF1(-), CD3(-), CD43(-), S-100(-), PCK(-), Syn(-), Ki-67(LI approximately 90%), EBER(-,positive control+). | M | 80 | IV | 3 | 1 | Fusion of BCL2 | CD79B  EP300  PIM1  IRF4  DTX1  TBL1XR1  ZFP36L1  BTG2 | | Adrenal gland | | 6R^2^ | Zanubrutinib | | | 0 |
| 22 | non-GCB | | CD20(+), CD19(+), PAX-8(+), BCL6(+), CD10(+), MUM1(slightly weak+), C-MYC(approximately 30%+), CD30(-), CD30(positive control)(+), CD5(-), BCL2(-), CyclinD1(-), CD3(-), CD43(-), CD34(-), TdT(-), GATA-3(-), UroplakinII(-), PCK(-), CK8/18(-), CK5/6(-), P40(-), P63(-), CK7(-), CK20(-), CD56(-), Syn(-), CgA(-), PSA(-), Nkx3.1(-), P504S(-), S-100(-), Ki-67LI: approximately 95%. | M | 80 | IV | 3 | 1 | Negative | MYD88  CCND3  B2M  BTG2  KMT2D  DTX1 | | Prostate | | 4R^2^ | Zanubrutinib | | | 0 |
| 23 | non-GCB | | CD20(+), CD3(-), CD10(-), Bcl6(+), MUM1(+), Bcl2(60%+), LMO2(-), CD19(+), CD22(+), PAX5(+), CD5(-), CyclinD1(-), CD21(-), CD30(-), CD38(partially+), TDT(-), MPO(-), C-Myc(60%+), Ki67(LI:80%), EBER(-). | F | 80 | IV | 3 | 1 | Fusion of BCL6 | MYD88  CD79B  NOTCH2  BTG1  PIM1  ARID1A  TBL1XR1  KMT2D  IRF4 | | Adnexal | | 4R^2^ | Zanubrutinib | | | 0 |
| 24 | GCB | | CD5(Tcell+),CD20(+++), CD79a (+) ,KI-67(80%+), CK-PAN (-),BCL-2 (approximately 80%+) ,BCL-6(approximately 50%+) ,CD10 (&GT: 30%+), CYCLIND-1 (-) ,CD21 (a small amount of residual FDC network+), CD23(-),MUM-1(slightly weak+),C-MYC (slightly weak+),CD3(Tcell+) | M | 56 | II | 1 | 0 | Negative | MYD88 | | - | | 6R-CHOP | | - | | - |
| 25 | non-GCB | | CD20(+),CD22(slightly weak +),CD19(slightly weak+),PAX5(+), CD10(-),Bcl6(-),MUM1(+),C-Myc(20%+), Bcl2(+),CD5(+), CyclinD1(-),CD3(-),Ki67(60%+). | F | 64 | II | 2 | 1 | Negative | CD79B  PIM1  BTG1 | | - | | 6R-CHOP | | - | | - |
| 26 | non-GCB | | Pax-5(+),CD20(+),CD79a(+), CD3(-),CD5(-), CyclinD1(-), SOX-11(-),CD21(-),CD10(-), Bcl-2(60%+),Bc1-6(+),Mum1(+), C-Myc(40%+). | F | 47 | IV | 2 | 0 | Negative | CD79B  CD70 | | Bone marrow | | 6R-CHOP | | - | | - |
| 27 | GCB | | CK(-),Vim(-),CD3 (scatteredly +),CD5 (scatteredly +),CD20(+), Pax-5(+),CD21(-), BCL-2(approximately 80%+), BCL-6(+),CD10(-),Mum1(-), CyclinD1(-),Ki-67 80%, ALK(-),CD30(-), CD43(+), S-100(-),CD99(+),TdT(-), Fli-1(-),EMA(-),Des(-), C-myc(approximately 40%). | F | 27 | IV | 3 | 1 | Negative | CD79B  MYD88 | | Bone marrow  Central nervous system | | 6R-CHOP | | - | | - |
| 28 | non-GCB | | Bcl-6(slightly weak +), BCL-2(50%+), CD15(-), CD20(+), CD21(FDC network +),CD3(+), CD30(-),C-MYC(approximately 40%+), CD45(-), EMA(-), Ki-67(+),MUM1(partially slightly weak +),Pax-5(+),TIA-1(-),CD2(-), CD4(-),CD56(-), CD7(-),CD8(-), CXCL-13(-),GRB(-), CD5(-), PD-1(-),CD10(-). | M | 61 | III | 3 | 0 | Negative | MYD88  KMT2D  BTG2  TBL1XR1  ARID1A | | - | | 6R-CHOP | | - | | - |
| 29 | non-GCB | | CD20(+),CD79a(+),MUM-1(+), BCL-2(approximately 70%+), BCL-6(approximately 70%+), C-MYC(approximately 40%+), PAX-5(+),CyclinD1(-),CD3(-), CD5(-),Ki-67(approximately 70%). | M | 69 | IV | 4 | 2 | Negative | CD79B  B2M | | Kidney | | 6R-CHOP | | - | | - |
| 30 | non-GCB | | CD20(+),CD79a(+),CD3(-),CD10(-),bcl-6(+),Mum -1(+), Cyclin D1(-), CD21(-),TdT(-),CD30(-),CD5(-), Bcl-2(+,70%~80%), C-Myc(+,60%~70%), P53(+,20%~30%),Ki-67(+,>80%). | M | 52 | IV | 2 | 1 | Negative | CD79B  B2M  PIM1  KMT2D  ARID1A  DTX1 | | Testis | | 6R-CHOP | | - | | - |
| 31 | non-GCB | | CD20(+),CD19(+),CD22(+), PAX5(+),CD5(+), BCL2(approximately 100%+), BCL6(approximately 70%+), Mum-1(approximately 90%+), C-MYC(approximately 60%+), CyclinD1(slightly weak +), CD43(slightly weak +),CD3(-), CD10(-),CD35(-),CD30(＜1% +), PCK (-),Ki67(Li approximately80%) | M | 43 | IV | 2 | 0 | Fusion of BCL6 | MYD88  CD79B  IRF4  KMT2D | | Bone marrow | | 6R-CHOP | | - | | - |
| 32 | GCB | | CD20(+,positive control+),CD19(+), CD22(+),PAX5(+),CD10(+), MUM1(+), BCL2(approximately 95%+), BCL6(approximately 20%+), C-MYC(approximately 40%+), CD43(partially +),CD23 and CD35(FDC network -), CyclinD1(-), SOX11(-),IgD(-),CD30(-), CD30(positive control)(+),CD5(-), CD3(-),TdT(-),CD34(-), MPO(-), Ki-67(LI approximately 90%). | M | 51 | IV | 3 | 2 | Negative | MYD88  KMT2D  EP300  IRF8  PIM1  BTG1  BTG2  DUSP2 | | Adrenal gland | | 6R-CHOP | | - | | - |
| 33 | non-GCB | | CD3(-),CD20(+),AE1/AE3(-), CK5/6(-),P40(-),EMA(-),P63(+/-), P16(-),EGFR(-),NUT(-),CD138(-), INI-1(+),S100(-),CD56(-), P53(+,>75%),Ki-67(+,95%), CD10(-), BCL6(partially+), MUM-1(+),ALK(-),CD21(-), CD23(-), CD30(-),CD5(-), CyclinD1(-), BCL2(+), c-myc(partially+), PD1(NAT,-), PDL1(22C3)(+,50%). | M | 60 | II | 2 | 1 | Negative | MYD88  CD79B  P1M1  BTG1  KMT2D | | - | | 6R-CHOP | | - | | - |
| 34 | non-GCB | | ALK(-)BCL2(+),BCL6(+),CD10(-),CD20(+),CD21(-),CD23(-),CD3(-), CD30(-),CD5(-),CyclinD1(-), Ki67(90%+),MUM-1(+),P53(+), c-myc(-). | M | 62 | IV | 4 | 2 | Negative | CD79B  EP300 | | Epityphlon | | 6R-CHOP | | - | | - |
| 35 | GCB | | CD3(-),CD20(+), Ki-67(approximately 70%+)，CD79a(+),c-myc(80%+), CK-pan(-), BCL2(-),BCL6(+),CD21(-), CD10(+),MUM1(-),CyclinD1(-), CD5(-), TDT(-). | F | 52 | III | 2 | 0 | Negative | CD79B  DTX1  EZH2  KMT2D | | - | | 6R-CHOP | | - | | - |
| 36 | non-GCB | | S100(-), KI67 80% GK(-), CD20(+),CD3(-), CyclinD1(-), CD10(-),BCL6(+),MUM1(+), CD30(-), c-myc(40%+), CD5(-), EBER(-), BCL2(approximately 60%+). | F | 54 | II | 1 | 0 | Negative | CD79B  B2M | | - | | 6R-CHOP | | - | | - |
| 37 | non-GCB | | CD3(background T cells＋), CD20 (+),CD21(-),Ki67 (75%+) , CD5(background cells＋), CD30(-), MUM-1(+), CD10 (-),BCL-6(+), BCL-2(+),CyclinD-1(-), P53 (30%+),C-MYC (30%+). | M | 47 | IV | 3 | 0 | Negative | MYD88  PIM1  SOCS1  IRF4  TNFAIP3 | | Testis  Bone  Heart | | 6R-CHOP | | - | | - |
| 38 | non-GCB | | Bcl2(+),Bcl6(-),CD10(-),CD19(+), CD20(+),CD23(+),CD3(-),CD5(-), CMYC(-),CYCLIND1(-),EBER(-), Ki-67(+60%),MUM-1(+). | M | 54 | IV | 3 | 1 | Negative | MYD88  KMT2D | | Kidney  Bone marrow | | 6R-CHOP | | - | |  |
| 39 | non-GCB | | CD20(L26) (+), CD20(positive control) (+),CD5(+), CD19(+),CD22(±）, AX-5(SP34)(partially+),CD79α(+), BCL-2(SP66) (+),CD3(2GV6) (-),CD43(-),CD7(-),CD21(±）, CD10(LN) (-),BCL-6(-),HGAL(-), CyclinD1(-), SOX11(-),IgD(-), CD23(-), CD35(-),LEF1(-),C-myc(-), Mum-1(-),CD30(-), P53(scatteredly +),TdT(-),CD34(-), CD99(-),MPO(-),CD1α(-), MCM2(LI approximately 50%), Ki-67(LI approximately 30-40%). | F | 45 | IV | 3 | 1 | Fusion of BCL6 | MYD88  CD79B  BTG2 | | Bone marrow | | 6R-CHOP | |  | |  |
| 40 | non-GCB | | CD23(-),CD20(++),PAX-5(+), CD19(+),CD3(scatteredly T cell+), CD5( scatteredly T cell +),CD10(-), BCL-6(+80%),MUM-1(+60%), BCL-2(-),CyclinD1 (-),TdT(-), P53(+50%),c-myc(+50%),CD30(-), CK-pan(-), Ki-67(+80%). | M | 55 | IV | 3 | 1 | Negative | MYD88  CD79B  EP300 | | Parotid gland  Epiglottis | | 6R-CHOP | |  | |  |

**NOTE:R-CHOP regimen**:Rituximab 375mg/m^2^, intravenously, day 1 before CHOP; Cyclophosphamide 750 mg/m^2^, IV drip, day 1; Vincristine 1.4mg/m^2^, maximum dose 2mg, intravenously, day 1; Doxorubicin 50 mg/m^2^, IV drip, day 1; Prednisone 60 mg/m^2^ Oral, day 1 to 21 days for a session, a total of 6 6 sessions of treatment.**R^2^ regimen**: Rituximab 375mg/m^2^, IV drip, day 1; Lenalidomide 10mg, orally, day 1 to 21,with aspirin for venous thrombosis prophylaxis. A 21-day course of treatment was used, a total of 6 sessions of treatment ( 2 elderly patients in the BTKi + R^2^ group discontinued the induction therapy after 4 sessions of treatment due to COVID-19 infection.Subsequent follow-up results were still CR).**Orelabrutinib** 150mg orally, once a day. **Zanubrutinib** 160mg orally, twice a day.
